# The Impact of Hydration on Metabolic Outcomes: From Arginine-Vasopressin Signaling to Clinical Implications

**DOI:** 10.3390/medicina61050838

**Published:** 2025-05-01

**Authors:** Andrijana Koceva, Andrej Janež, Mojca Jensterle

**Affiliations:** 1 Department of Endocrinology and Diabetology, Clinic of Internal Medicine, University Medical Center Maribor, 2000 Maribor, Slovenia; 2 Faculty of Medicine, University of Maribor, 2000 Maribor, Slovenia; 3 Department of Endocrinology, Diabetes and Metabolic Diseases, University Medical Center Ljubljana, 1000 Ljubljana, Slovenia; 4 Faculty of Medicine, University of Ljubljana, 1000 Ljubljana, Slovenia

**Keywords:** arginine-vasopressin (AVP), hydration, glucose regulation, lipid metabolism, metabolic outcomes

## Abstract

Arginine-vasopressin peptide (AVP) plays a critical role in water balance and osmoregulation. However, emerging evidence suggests that AVP’s actions may expand beyond its traditional role, significantly influencing metabolic regulation, including glucose homeostasis, insulin sensitivity, lipid metabolism and energy balance. Elevated AVP levels are seen in various metabolic conditions, such as insulin resistance, metabolic syndrome, type 2 diabetes (T2D) and obesity, further highlighting its potential role as a metabolic regulator. As AVP levels are regulated by hydration status, studies have proposed that chronic hypohydration and persistently elevated AVP levels may contribute to metabolic dysfunction, where increased hydration and therefore AVP suppression may lead to potential metabolic improvements. By analyzing data from animal studies, human observational research and interventional trials, this review evaluates the current evidence on the potential causal relationships and impact of AVP on metabolic regulation, as well as exploring the role of hydration in AVP-mediated metabolic outcomes.

## 1. Introduction

Hydration is widely recognized as being essential for overall health [1], yet specific recommendations for daily water intake remain limited in most metabolic disease guidelines. This omission persists despite emerging evidence suggesting that adequate hydration may play a role in managing metabolic conditions such as type 2 diabetes, metabolic syndrome and obesity, and that inadequate hydration may represent a risk factor for new-onset hyperglycemia [2,3].

Arginine-vasopressin peptide (AVP), a nine amino-acid peptide hormone, plays a crucial role in body fluid regulation [2]. It is produced mainly in the parvocellular and magnocellular neurons located in the supraoptic and paraventricular nucleus of the hypothalamus as a response to high plasma osmolality, decreased plasma volume or low blood pressure [2,4]. AVP has a short half-life, as more than 90% of the released AVP is bound to platelets and inactivated in the liver and kidney [5]. Copeptin, on the other hand, derives from the AVP precursor and is synthesized in a one-to-one ratio with AVP, which makes it a metabolically stable surrogate marker of AVP production [6]. AVP maintains body fluid balance by increasing water reabsorption and regulates blood pressure by inducing vasoconstriction; however, there is emerging evidence of AVP’s involvement in metabolic regulation, including glucose homeostasis, insulin sensitivity, lipid metabolism and energy balance [7]. Dysregulated AVP signaling, characterized by elevated AVP or its surrogate marker copeptin, has been implicated in the development of various metabolic disorders. Conversely, increased hydration, potentially by lowering AVP secretion, has been associated with improved metabolic outcomes [2,7].

While previous reviews have focused either on the physiological roles of AVP in glucose and lipid metabolism or on the benefits of hydration on metabolic health [1,2,6,7,8,9], our narrative review aims to provide a more integrated perspective on this complex interplay. We began by outlining the fundamental mechanisms of AVP signaling, followed by a discussion of its distinct metabolic roles based on insights from receptor-specific knockout studies. We then aimed to expand the scope by examining associations between circulating AVP (and its surrogate marker copeptin) and a range of metabolic disorders, including the relationship between AVP and metabolic dysfunction-associated steatotic liver disease (MASLD) and AVP and cardiovascular health. We also explored the potential metabolic implications of hypophysitis and AVP deficiency syndrome. Finally, we considered how hydration status modulates AVP activity and, subsequently, impacts metabolic outcomes. This integrated approach highlights hydration as a potentially modifiable factor in the prevention and management of metabolic diseases.

We conducted a comprehensive search using PubMed with advanced search strategies of relevant keywords such as “AVP”, “arginine vasopressin”, “copeptin”, “hydration”, “hypohydration”, “water intake”, “hypophysitis”, “diabetes insipidus” and “AVP deficiency” in combination with “metabolic regulation”, “metabolic outcomes”, “glucose metabolism disorders”, “glucose regulation”, “lipid metabolism”, “steatosis” “cardiovascular diseases”, “cardiovascular outcomes”, “hypertension”, “heart failure” and “myocardial infarction”. Articles were selected based on relevance, peer-reviewed status and focus on either physiological or disease-related aspects of AVP signaling in metabolic contexts. We included animal mechanistic studies, clinical observational data and interventional trials. To identify further relevant studies and ensure a comprehensive understanding of the current state of research, additional references were identified through manual screening and reviewing article reference lists.

## 2. Vasopressin (AVP) Signaling: Mechanisms and Pathways

AVP exerts its physiological effects by acting on three subtypes of AVP receptors, V1a receptor (V1aR), V1b receptor (V1bR) and V2 receptor (V2R), all of which are part of the G-protein coupled receptor family [10]. By binding to V2 receptors in the collecting ducts of the kidney, AVP signals an increased distribution of aquaporin to the luminal membrane, thereby increasing water reabsorption [9]. Apart from this most known AVP function of regulating fluid homeostasis, AVP also regulates body vasoconstriction and modulates adrenocorticotropic hormone (ACTH) release [10]. Additionally, evidence shows that AVP signaling may be important in metabolic regulation as well, as AVP receptors are expressed in several organs implicated in glucose and lipid metabolism. V1aR are expressed in the liver, V1bR are found in the pancreas, adipose tissue and pituitary, and both V1aR and V1bR are found in the adrenal glands, while V2R, apart from being found in the kidney, are also expressed in the heart, liver, muscle and white and brown adipose tissue, all of which are insulin-sensitive tissues [6].

AVP contributes to metabolic regulation by two mechanisms: directly, by acting on the liver and pancreas, and indirectly, by stimulating the hypothalamic-pituitary-adrenal axis [2].

AVP action on hepatic V1aR increases hepatic glucose production by stimulating glycogenolysis as well as gluconeogenesis, while pancreatic V1bR activation induces insulin secretion at times of high plasma glucose levels and glucagon secretion at times of low plasma glucose levels [6]. A recent study also revealed AVP-mediated crosstalk between pancreatic α and β cells [11]. By evaluating AVP-mediated insulin secretion from isolated mouse islets, the researchers found that AVP stimulates glucagon secretion by activating V1bR on α cells. The released glucagon then activates glucagon-like peptide-1 (GLP-1) receptors on β cells, enhancing glucose-dependent insulin secretion [11]. This AVP-induced insulin secretion is completely blocked by the co-injection of the GLP-1 receptor antagonist Exendin (9–39), which confirms that the insulinotropic effect of AVP may be mediated by paracrine pathways involving GLP-1 receptors [11]. AVP has also been shown to stimulate GLP-1 secretion by activating V1bR receptors on L-cells in the colon [12]. Additionally, GLP-1 receptor agonists are known to enhance the antidiuretic effects of desmopressin in patients with AVP deficiency, additionally confirming that AVP and GLP-1 may have bidirectional positive feedback interaction [13].

AVP also regulates glucose metabolism through its modulation of the hypothalamic-pituitary-adrenal (HPA) axis. By activating V1bR in the pituitary gland, AVP directly stimulates ACTH synthesis, which leads to increased cortisol production [6]. Additionally, AVP activation of V1aR in the adrenal cortex further enhances cortisol release, while V1bR activation in the adrenal medulla promotes epinephrine secretion. Both cortisol and epinephrine have an impact on hepatic glucose production and insulin sensitivity [14].

AVP is also implicated in appetite and food intake regulation, as it may suppress the neuropeptide Y-induced orexigenic effect by V1aR signaling [15]. Lastly, AVP binding to V1aR in the smooth muscles of the gastrointestinal wall increases contractions and regulates gastrointestinal (GI) motility, which also impacts food absorption [16].

**Figure 1:**
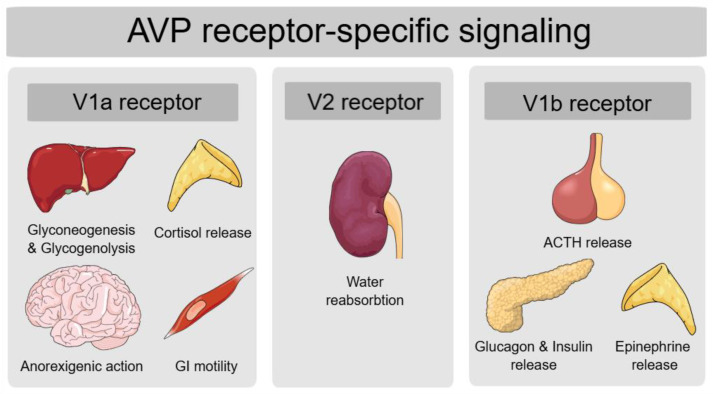
AVP functions on metabolic regulation according to its receptor-specific signaling. Legend: AVP—arginine vasopressin peptide; V1a—vasopressin 1a; V2—vasopressin 2; V1b—vasopressin 1b; GI—gastrointestinal; ACTH—adrenocorticotropic hormone.

In Figure 1, we summarize the physiological actions of AVP based on its receptor subtypes.

## 3. AVP Impact on Metabolic Regulation: Insights from Knockout Studies

In wild-type animals, AVP enhances gluconeogenesis and glycogenolysis, increases lipoprotein lipase (LPL) activity and stimulates hepatic lipogenesis [7]. Various animal studies using vasopressin receptor knockout provide further evidence of the implication of AVP and AVP receptors in modulating glycemic and lipid metabolism.

V1aR-deficient mice have enhanced lipid metabolism and ketone body production by triglycerides and free fatty acid metabolism and beta-oxidation, which indicates that in physiological conditions, V1aR signaling suppresses lipid metabolism and ketogenesis [17]. Insulin-induced Akt phosphorylation is also reduced in the adipocytes of V1aR-deficient mice. This reduced glucose uptake in adipose tissue results in insulin resistance and altered glucose tolerance [17]. V1aR-deficient mice fed with a normal-chow (NC) diet show impaired glucose tolerance, insulin resistance and up-regulation in hepatic glucose production; however, hyperglycemia and hyperleptinemia in V1aR-deficient mice fed with NC diet leads to hypophagia and decreased weight gain compared to controls [18]. In contrast to V1aR-deficient mice fed with an NC diet, V1aR-deficient mice fed with a high-fat (HF) diet experience significantly increased calorie intake, leading to obesity and a shift from prediabetes to diabetes, which possibly results from hyperleptinemia in the presence of leptin resistance [18]. V1aR-deficient mice also have decreased levels of aldosterone, which decreases plasma volume and may also exacerbate hyperglycemia [18].

On the other hand, V1bR-deficient mice also have altered glucose metabolism, as they exhibit lower glucose, insulin and glucagon levels, showing enhanced insulin sensitivity and increased insulin-induced phosphorylation of Akt in adipocytes. Lower glucose levels are likely due to increased insulin sensitivity, reduced glucagon levels and/or reduced ACTH and corticosterone levels [19]. Additionally, V1bR-deficient mice experience enhanced insulin sensitivity under both the NC diet as well as HF diet [10]. This shows that in physiological conditions, V1bR activation likely contributes to metabolic dysfunction.

Double V1aR/V1bR-deficient mice, similarly to V1aR-deficient mice, show higher insulin levels, decreased insulin sensitivity, reduced insulin-induced Akt phosphorylation in the adipocytes and higher glucose levels under both diets, indicating impaired glucose tolerance and insulin resistance [10]. This impairment in glucose tolerance observed in the setting of V1aR deficiency, regardless of concurrent V1bR deficiency, indicates that AVP has a greater influence on glucose regulation through V1aR signaling rather than V1bR signaling.

**Figure 2:**
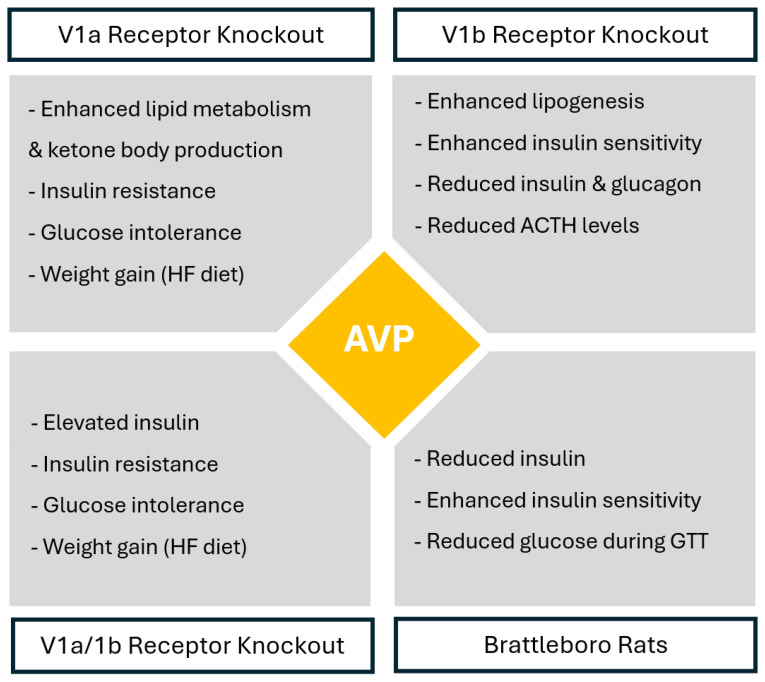
AVP-mediated receptor-specific metabolic regulation based on receptor knockout studies. Legend AVP—arginine vasopressin peptide; HF diet—high-fat diet; GTT—glucose tolerance test; ACTH—adrenocorticotropic hormone.

Lastly, based on the findings from these knockout studies, Brattleboro rats—genetically lacking AVP and widely used as a model for studying central AVP deficiency—are expected to display glucose-intolerant phenotype [20]. Nevertheless, Brattleboro rats show enhanced glucose tolerance, with lower insulin levels, low glucose levels during glucose tolerance tests and enhanced insulin sensitivity compared to wild-type rats, which suggests that AVP acts on glucose metabolism not only by V1aR/V1bR signaling but also by altering the water homeostasis through V2R signaling [20]. Figure 2 summarizes AVP functions based on different receptor deficiencies.

## 4. AVP Impact on Metabolic Regulation: Insights from Animal and Human Studies

### 4.1. AVP and Diabetes

AVP involvement in glucose metabolism has been additionally explored by chronically altering AVP in both directions, either by AVP infusion (high AVP condition) or increasing water intake (low AVP condition). Chronically high AVP levels induce hyperglycemia in healthy nonobese rats as well as aggravate glucose tolerance and insulin resistance in obese rat models, while treatment with V1aR antagonist reverses these changes [21,22]. Similarly, in a study by Spruce et al., AVP infusion in healthy adults increased glucose, with a concomitant increase in glucagon levels but not insulin levels [23]. Another crossover trial by Jansen et al. also confirmed that increasing plasma osmolality and copeptin levels by infusion of 3% hypertonic NaCl resulted in greater hyperglycemic responses during an oral glucose tolerance test, possibly due to greater glucagon levels [24]. While AVP infusions are connected to hyperglycemia, in a study by Taveau et al. on obese Zucker rats, lowering of AVP levels by increased water intake had no efficacy on glucose tolerance but did revert hepatosteatosis [21].

Population-based studies have identified several single nucleotide polymorphisms in the AVP, V1aR and V1bR genes that are associated with insulin resistance, hyperglycemia, obesity and diabetes development [7]. Additionally, epidemiological and clinical studies have also suggested the connection between elevated AVP levels and the risk of hyperglycemia, diabetes and metabolic syndrome development [6]. Several large longitudinal cohorts investigated the association of AVP or copeptin with the risk of diabetes development [25,26,27,28]. Enhörning et al. analyzed the association of increasing copeptin levels with the risk of new-onset type 2 diabetes (T2D) development in a Swedish population-based sample of 4742 subjects and found that elevated copeptin levels predicted diabetes independently of various diabetes risk factors, including fasting glucose and insulin levels [25]. Roussel et al. investigated the association between plasma copeptin with insulin sensitivity and the risk of impaired fasting glucose (IFG) and T2D development in 5110 subjects. Their findings showed that elevated copeptin levels were associated with decreased insulin sensitivity and a higher incidence of IGF/T2D [26]. Certain single nucleotide polymorphisms within the AVP 2 gene were connected to both high AVP levels and high incidence of IFG in males, but not in females [26]. Wannamethee et al. examined a British cohort of 3226 older males and confirmed the association of copeptin with an increased risk of diabetes in older men [27]. On the other hand, Abbasi et al. examined the usefulness of copeptin levels for the prediction of T2D development in a Dutch cohort of males and females separately, showing a stronger association and predictive value of plasma copeptin levels with the risk of T2D in females than in males [28].

AVP also plays an important role in regulating glucagon secretion during hypoglycemia. Clamp studies have demonstrated that under hypoglycemic conditions, there is a significant increase in AVP, copeptin and glucagon levels, where these elevations are not observed during euglycemia [4]. Moreover, this hypoglycemia/insulin-induced copeptin and glucagon elevation is notably attenuated in individuals with type 1 diabetes (T1D) compared to non-diabetic BMI and age-matched controls [4]. Additionally, hypoglycemia/insulin-induced copeptin elevation is associated with hypoglycemia awareness in T1D [29]. This implies that copeptin monitoring may serve as a biomarker for assessing and stratifying hypoglycemia risk in patients with T1D [4].

Similarly, as in previous research [9], we were unable to find data regarding metabolic health in patients with hypophysitis or central AVP deficiency (previously called central diabetes insipidus) with the exception of a case series by Crowley et al. describing 13 patients with adipsic form of central AVP deficiency [30]. In this cohort, 8 out of 13 patients were classified as obese, 3 were overweight and 7 had sleep apnea. Despite these findings, only one patient had diabetes mellitus and only one patient had hyperlipidemia—both of whom also had panhypopituitarism. In fact, 8 out of the 13 patients were also diagnosed with panhypopituitarism, which may have significantly influenced their metabolic profile [30]. Therefore, the extent to which AVP deficiency per se contributes to metabolic alterations in this population remains unclear. Based on findings from AVP-deficient Brattleboro rats, AVP deficiency would most likely be associated with a lower prevalence of glucose intolerance or T2D. Nevertheless, despite being considered rare, the coexistence of central AVP deficiency and T2D has been described in a few case reports [31,32,33].

Desmopressin, a synthetic analog of AVP, primarily targets V2R to promote antidiuretic effects in patients with AVP deficiency. Clinical evidence from patients with central AVP deficiency showed that desmopressin administration increases insulin-like growth factor-binding protein 1 (IGFBP-1) without altering IGF-1, glucose, insulin, glucagon or cortisol levels [34]. Similarly, in AVP-deficient Brattleboro rats with streptozotocin-induced diabetes, desmopressin infusion normalized water consumption and body weight, yet had no impact on diabetes-associated alterations of the HPA axis or glucose metabolism [35]. These findings suggest that while desmopressin is effective in restoring fluid balance in the context of central AVP deficiency, its role in modulating glucose metabolism remains limited and requires further investigation.

### 4.2. AVP and Metabolic Syndrome

Insulin resistance and high insulin levels increase insulin-regulated aminopeptidase (IRAP) activity, which accelerates AVP degradation and triggers a compensatory rise in AVP synthesis, potentially contributing to the development of metabolic syndrome [36]. Moreover, fructose intake has been shown to stimulate AVP secretion to a greater extent than glucose [37], with high AVP levels in fructose-fed mice leading to the development of metabolic syndrome via V1bR activation. Interestingly, fructose-fed V1bR-deficient mice, despite having elevated copeptin levels, are protected from the development of metabolic syndrome, highlighting a receptor-specific mechanism of AVP signaling. Additionally, increased water intake in fructose-fed mice lowers AVP secretion, which, in turn, prevents or ameliorates fructose-induced metabolic syndrome [38].

Several cross-sectional and observational studies have connected elevated AVP or copeptin levels to multiple elements of the metabolic syndrome. According to a cross-sectional study of 4742 subjects, copeptin levels were associated with hypertension, abdominal obesity, obesity, high fat intake, low physical activity and the development of metabolic syndrome [39]. In an observational study consisting of 2599 individuals from the Malmö Offspring Study (MOS), Brunkwall et al. found that high copeptin levels were positively associated with all metabolic traits (glycated hemoglobin (HbA1c), glucose, triglycerides, body mass index (BMI) and waist circumference), showing an association between high AVP and dysregulated glucose and lipid metabolism [40]. Saleem et al. examined the association of copeptin levels with insulin resistance and metabolic syndrome by cross-sectional analysis of two cohorts: 1293 African American subjects and 1197 non-Hispanic white subjects. In both cohorts, high copeptin levels were associated with the presence of an increasing number of metabolic syndrome elements, such as high waist circumference, hypertension, hyperglycemia, hypertriglyceridemia and reduced HDL cholesterol [41]. By re-examining subjects from the population-based Malmo Diet and Cancer Study cardiovascular cohort, Enhörning et al. showed that increasing copeptin levels were associated with an increasing incidence of abdominal obesity, T2D and microalbuminuria, suggesting a relationship between AVP and cardiometabolic risk [42]. Wannamethee et al. examined 3226 older males without baseline diabetes from the British Regional Heart Study and showed a significant positive association of copeptin levels with renal dysfunction, insulin resistance, a cluster of cardiometabolic risk factors (waist circumference, hypertension, abdominal obesity, atherogenic dyslipidemia), inflammation (c-reactive protein) and endothelial dysfunction (von Willebrand factor, tissue plasminogen) [27].

### 4.3. AVP and Obesity

AVP may demonstrate important metabolic effects that impact obesity pathophysiology. Animal studies have shown that central leptin administration increases AVP levels [43] and that AVP suppresses calorie intake, predominantly by acting on V1aR [15,44,45]. Copeptin levels are higher in obesity, with males having higher overall levels compared to females [46,47].

### 4.4. AVP, Dyslipidemia and Hepatosteatosis

AVP also plays a role in lipid metabolism. In healthy individuals, administration of V1aR agonists such as lysine-vasopressin has been associated with a reduction in circulating non-esterified fatty acids, indicating inhibition of adipose tissue lipolysis [48]. Patients with central AVP deficiency (previously referred to as central diabetes insipidus) also exhibit significant dysregulation of lipid metabolism, characterized by hypertriglyceridemia and, in some cases, abnormal lipoprotein profiles. Treatment with the V2R agonist desmopressin in these patients has been associated with increased lipoprotein lipase activity and a reduction in triglyceride levels, accompanied by the disappearance of abnormal lipoproteins [49]. Conversely, the use of a V2R antagonist tolvaptan in patients with polycystic kidney disease has been linked to increased total cholesterol and low-density lipoprotein (LDL) levels, suggesting V2R-mediated AVP actions on lipid metabolism [7,50].

Metabolic dysfunction-associated steatotic liver disease (MASLD) is the most common chronic liver disease, with an estimated prevalence of around 25% in the general population and 80% in individuals with obesity [51]. Barchetta et al. examined the relationship between plasma copeptin levels and the presence and severity of MASLD and metabolic dysfunction-associated steatohepatitis (MASH) in 60 individuals with obesity undergoing bariatric surgery. All subjects had biopsy-proven MASLD/MASH and were compared to healthy non-obese controls. The group confirmed that higher copeptin levels were independently associated with both the presence and severity of MASLD/MASH. Specifically, copeptin levels were greater in obese individuals with MASLD compared to both obese individuals without MASLD and non-obese healthy controls [51]. Additionally, copeptin levels increased in parallel with MASH severity, suggesting a potential role of AVP signaling in liver disease progression [51]. The relationship between copeptin and the presence and severity of MASLD in obesity was also investigated by Majumdar et al., who also confirmed higher copeptin levels in patients with both obesity and MASLD compared to those with obesity alone. Similarly, copeptin levels were positively associated with increased MASLD severity [52].

The involvement of AVP in MASLD and hepatic steatosis is multifaced. Elevated AVP levels have been associated with enhanced hepatic gluconeogenesis and glycogenolysis, contributing to hyperglycemia and the development of insulin resistance—both key drivers of hepatic lipid accumulation. In addition, AVP influences lipid metabolism directly by promoting lipogenesis and suppressing lipolysis, thereby promoting triglyceride accumulation within hepatocytes, a hallmark feature of hepatic steatosis [51,53,54].

### 4.5. AVP and Cardiovascular Health

Since AVP is a key regulator of water balance and vascular tone, its levels are altered in various cardiovascular diseases. There is a positive association between copeptin and the development of hypertension [55]. Copeptin levels are also higher in patients with resistant hypertension [56,57]. The proposed mechanisms underlying the role of AVP in the development of hypertension include AVP production induced by the activation of the renin-angiotensin-aldosterone system, vasoconstriction mediated by the direct effects of AVP on smooth muscle cells and indirectly through renin secretion, as well as AVP-induced increased tubular sodium retention [55].

Elevated AVP levels have been observed in patients with congestive heart failure (CHF) and correlate with disease severity [58,59]. AVP contributes to both systolic and diastolic dysfunction by V1a-induced vasoconstriction, which increases afterload and V2-induced water retention [59]. Additionally, by V1a-mediated regulation of beta-adrenergic receptor responsiveness, AVP has been associated with impaired cardiac contractility [60]. Therefore, increased AVP may worsen cardiac function as well as being a marker of the presence and severity of CHF and a predictor of long-term clinical outcomes [58,59,61].

A population-based longitudinal study has also shown that elevated copeptin levels are independently connected to increased risk of coronary artery disease development in individuals with and without diabetes [62]. Additionally, copeptin strongly predicted cardiovascular mortality with subjects in the highest copeptin quartile, who showed a >70% increased risk of dying from cardiovascular disease compared to subjects in the lowest copeptin quartile [62]. Copeptin has also been associated as a predictor for higher mortality and major cardiovascular events in patients with known coronary artery disease [63], as well as being strongly associated with stroke, sudden death, combined cardiovascular events and all-cause mortality in patients with end-stage renal disease with or without type 2 diabetes [64,65].

In acute myocardial infarction, AVP levels, as indicated by copeptin, are elevated and have been associated with coronary microvascular dysfunction [66]. High copeptin levels after AMI are linked to prognostically worse outcomes, such as larger infarct sizes, post-infarction left-ventricular dysfunction and cardiac remodeling [67,68], indicating that they can serve as a predictor for major cardiac adverse events [69,70] as well as a predictor for mortality and morbidity in patients with CHF after AMI [71]. Copeptin levels were also found to be an independent, time-sensitive marker of the post-cardiac arrest syndrome severity and predictor of early mortality [72].

## 5. The Impact of Hydration on AVP and Metabolic Outcomes

### 5.1. Hypohydration and Metabolic Outcomes

Hyperglycemia, a hallmark of diabetes, presents with certain challenges to the body water balance [2]. Due to its osmotic effects, hyperglycemia drives water movement from the intracellular compartment to the extracellular space, preserving plasma osmolality at the expense of cellular dehydration. Furthermore, glucosuria leads to increased urinary water losses, further increasing the risk of dehydration [2]. To compensate for these fluid shifts and maintain vascular volume and osmolality, diabetes is therefore associated with an elevation of AVP levels [2]. Conversely, alterations in body hydration may also negatively impact glucose regulation, highlighting the bidirectional relationship between fluid balance and metabolic outcomes [73,74].

Keller et al. investigated the effect of acute hyper-, iso- and hypo-osmolality on body protein, glucose and lipid metabolism in 10 healthy male volunteers. The group showed that hyperosmolality induced glycogenolysis while hypo-osmolality promoted lipolysis combined with glucose and protein-sparing effect [73]. Another small interventional study of males with T2D by Johnson et al. also showed that three-day water restriction or hypohydration, compared to euvolemic conditions, led to impaired glucose response, elevated cortisol during an oral glucose tolerance test, impaired insulin sensitivity, insulin resistance and reduced whole-body glucose disposal. Notably, these effects occurred without activation of the renin-angiotensin-aldosterone system, suggesting a potential causal role of AVP and hydration status in glucose regulation [74]. In contrast to previous studies, a small, randomized crossover trial by Carroll et al. which included 16 healthy adults confirmed that although hypohydration increased copeptin levels and reduced muscle cross-sectional area and muscle water content, acute mild hypohydration did not result in measurable changes in glucose metabolism, which suggests that short-term fluid deficits may not be sufficient to disrupt glycemic control in healthy individuals [9].

### 5.2. Impact of Increased Hydration on Metabolic Outcomes

Since high AVP and copeptin levels have been associated with poorer cardiometabolic health, and water intake is known to lower copeptin levels [75], in order to improve metabolic outcomes, past research focused on interventions aimed at reducing copeptin, mainly by increasing water intake. In a cross-sectional study using an online survey of 138 adults by Carroll et al., water intake was negatively correlated with T2D risk [76]. Roussel et al. examined the association between self-declared water intake and the risk for impaired fasting glucose and diabetes during a nine-year follow-up of 3615 participants from a prospective cohort in the DESIR study [3]. The group found that water intake was negatively associated with the risk of new-onset hyperglycemia and subjects consuming 500 to 1000 mL of water a day had a 32% lower risk of hyperglycemia compared to those consuming less than 500 mL of water a day [3]. In an observational study consisting of 2599 individuals from the MOS Study, Brunkwall et al. found a significant negative association between increasing water intake and triglyceride levels and a positive association with HDL cholesterol levels [40]. Although increased water intake showed no association with fasting glucose levels or HbA1c, increasing urine osmolality was associated with elevated fasting glucose and borderline significant with elevated HbA1c [40].

Some studies have suggested a possible sex-specific difference in the impact of hydration on glucose control. In a sex-stratified, cross-sectional analysis of the UK National Diet and Nutrition Survey, Carroll et al. evaluated the association between water intake and glycated hemoglobin (HbA1c) in 456 men and 579 women and found that one cup (240 mL) of water per day was associated with a 0.04% lower HbA1c and a 22% reduced likelihood of HbA1c > 5.5% in males, while no association was found in females [77]. This sex-specific difference may also be implied in the study by Pan et al., where in a large cohort consisting of 82,902 young and middle-aged women, no overall association between water intake and T2D risk was found. However, the substitution of sugar-sweetened beverages and fruit juices with water did result in a modestly lower risk for T2D [78].

Other studies evaluated the impact of hydration on weight change [79,80]. A randomized interventional trial of 48 individuals by Dennis et al. investigated the impact of increased water consumption combined with a hypocaloric diet on weight loss. Adding 500 mL of water before main meals in the interventional group led to greater weight loss over 12 weeks compared to diet alone. Additionally, the interventional group experienced a 44% greater decline in weight, although this may be partly explained by the acute reduction in meal energy intake following water ingestion [79]. The effects of pre-meal water intake on copeptin, glucose metabolism, lipid regulation and anthropometric indices were also examined in a randomized study of 40 patients with T2D by Sedaghat et al. Increasing pre-meal water intake over eight weeks reduced copeptin, fasting glucose, triglycerides, LDL levels, waist circumference and BMI [80].

Some studies have suggested that the metabolic benefits of hydration-induced AVP reduction may depend on baseline AVP levels [81]. In a randomized controlled study by Nakamura et al., increased water intake over 12 weeks in healthy individuals lowered systolic blood pressure and raised body temperature but had no significant impact on fasting glucose—likely due to low baseline AVP levels in the intervention group [81]. The association between water intake and hypertension was also investigated by Li et al. in a large-scale longitudinal observational cohort study. Their findings revealed an inverse association between water consumption and the risk of developing hypertension over a nine-year follow-up period [82].

In a study by Enhörning et al. examining the acute and medium-term effects of hydration on glucose regulation in 37 healthy volunteers, acute water intake (1 L in 20 min) reduced copeptin levels by 39%, while a 3-L daily water intake increase over one week led to a 15% copeptin reduction compared to only habitual water intake [83]. Nevertheless, in subjects with a good intervention response, indicated by the greatest decline in copeptin, increased hydration over one week did not change plasma glucose or insulin levels but did significantly reduce glucagon levels, which aligns with AVP’s role in stimulating glucagon secretion by activating V1bR on pancreatic α cells [83]. Based on these results, the group proposed that future interventional studies aimed at investigating the impact of hydration on cardiometabolic outcomes by copeptin suppression should prioritize individuals with high baseline copeptin levels and low habitual water intake, as they may be more responsive to hydration-based interventions [83]. In a subsequent six-week interventional study, Enhörning et al. increased hydration (1.5 L of water beyond habitual intake) in subjects considered as potential responders (habitual low-drinkers with high baseline copeptin). This resulted in a significant copeptin reduction and a small but significant fasting glucose reduction, without a change in insulin or glucagon levels [84]. Similar to the previous study [83], despite subjects being instructed to drink 1.5 additional liters of water, the achieved mean difference in self-reported daily water intake was approximately 900 mL, showing that increased hydration reduces habitual water intake [84]. A currently ongoing, large long-term randomized controlled trial (NCT03422848) will determine whether increasing hydration for 12 months in subjects with low habitual water intake can reduce fasting glucose and the risk of new-onset diabetes and other cardiometabolic risk factors [84]. Table 1 summarizes studies investigating the effect of increased hydration on various metabolic outcomes.

## 6. Conclusions

Beyond its well-established role in maintaining fluid balance, AVP has also been recognized as a key regulator of metabolic pathways implicated in glucose and lipid metabolism. Dysregulation in AVP signaling and elevated AVP levels have been linked to the development of various metabolic disorders. Conversely, as hydration appears to be a key regulator of AVP secretion, accumulating evidence supports the role of adequate water intake in improving metabolic parameters through the modulation of AVP signaling. Therefore, from a clinical perspective, hydration may be a promising yet underrecognized, modifiable factor in the management of metabolic diseases. Integrating hydration-focused interventions into clinical practice may offer a safe, accessible and cost-effective complement to conventional therapies, warranting greater attention in both research and clinical guideline development. Further research should focus on clarifying the mechanisms underlying AVP’s metabolic effects and determining whether targeting AVP signaling, possibly through modulating water intake, could serve as an effective strategy for the prevention and management of metabolic diseases.

## Figures and Tables

**Table 1:** Summary of studies examining the effects of hydration on metabolic outcomes.

Study Author	Objective	Study Design and Participants	Findings
Keller et al., 2003 [73]	Effect of hyper-, iso- and hypo-osmolality on protein, glucose and lipid metabolism.	Randomized crossover trial; 10 healthy males;	Hyperosmolality induced glycogenolysis while hypo-osmolality promoted lipolysis with glucose and protein-sparing effect.
Johnson et. al., 2017 [74]	Effect of reduced water intake over 3 days on glucose regulation in T2D.	Randomized crossover trial; 9 males with T2D;	Reduced water intake acutely impaired glucose regulation.
Carroll et al., 2019 [9]	Effect of acute hypohydration compared to euhydration on glucose regulation.	Randomized crossover trial; 16 healthy adults;	Acute mild hypohydration led to an increase in copeptin without a change in glucose regulation.
Carroll et al., 2015 [76]	Association between water intake and T2D risk.	Observational (cross-sectional); Online survey 138 participants;	Water intake was negatively associated with T2D risk score.
Roussel et al., 2011 [3]	Association between self-declared water intake and the risk for IFG/diabetes.	Observational longitudinal (cohort) study; 3615 participants;	Water intake was negatively associated with the risk of new-onset hyperglycemia.
Carroll et al., 2016 [77]	Association between water intake and HbA1c.	Sex-stratified, cross-sectional analysis; 456 men and 579 women;	Higher water intake was associated with lower HbA1c in males, but not in females.
Pan et al., 2012 [78]	Association of water intake and T2D incidence.	Observational (cohort) study; 82,902 women;	Water intake was not associated with the risk of T2D.
Nakamura et al., 2020 [81]	Effect of increasing water intake over 12 weeks on fasting blood glucose levels and hydration status.	Randomized controlled trial; 57 healthy Japanese subjects;	Increased water intake decreased systolic blood pressure and increased body temperature, without an impact on fasting glucose.
Dennis et al., 2010 [79]	Effect of pre-meal water consumption over 12 weeks on weight loss.	Randomized controlled trial; 48 individuals with overweight or obesity;	Pre-meal consumption of water led to greater weight loss compared to a hypocaloric diet alone.
Brunkwall et al., 2020 [40]	Association between high water intake, low urine osmolality and metabolic profile.	Observational (cross-sectional) study; 2599 participants;	High water intake was associated with a favorable lipid profile.
Sedaghat et al., 2021 [80]	Effects of pre-meal water intake over 8 weeks on copeptin, glucose and lipid regulation, and anthropometric indices.	Randomized controlled trial; 40 patients with T2D;	Pre-meal water intake was associated with a reduction in copeptin, fasting glucose levels, triglycerides, LDL, waist circumference and BMI.
Li et al., 2024 [82]	Association between water intake and risk of hypertension	Longitudinal observational study; 3823 participants;	Inverse trend between water intake and the risk of hypertension development.
Enhörning et al., 2019 [83]	Acute and medium-term effects of increased water intake over 1 week on copeptin levels and glucose.	Randomized crossover trial; 37 healthy volunteers;	Increased water intake decreased copeptin levels, did not change plasma glucose levels but reduced fasting glucagon.
Enhörning et al., 2019 [84]	Effects of increased water intake over 6 weeks on copeptin and fasting glucose levels in potential responders.	Pilot interventional trial; 31 healthy volunteers with high copeptin;	Water supplementation in subjects with habitually low water intake reduced both copeptin and fasting glucose levels.

Legend: T2D—type 2 diabetes; IFG—impaired fasting glucose; HbA1c—glycated hemoglobin; LDL—low-density lipoprotein; BMI—body mass index.

## Data Availability

No new data was created in this article; Data sharing is not applicable.

## Abbreviation

AVP	arginine-vasopressin peptide;
T2D	type 2 diabetes;
V1aR	vasopressin 1a receptor;
V1bR	vasopressin 1b receptor;
V2R	vasopressin 2 receptor;
ACTH	adrenocorticotropic hormone;
GI	gastrointestinal;
NC diet	normal chow diet;
HF diet	high-fat diet;
GTT	glucose tolerance test;
T1D	type 1 diabetes;
IGF BP-1	insulin-like growth factor-binding protein 1
IRAP	insulin-regulated aminopeptidase
MASLD	metabolic dysfunction-associated steatotic liver disease;
MASH	metabolic dysfunction-associated steatohepatitis;
CHF	congestive heart failure;
AMI	acute myocardial infarction;
DESIR	Data from Epidemiological Study on the Insulin Resistance Syndrome
MOS	Malmö Offspring Study
IFG	impaired fasting glucose;
HbA1c	glycated hemoglobin;
LDL	low-density lipoprotein;
BMI	body mass index;
